# *Peribacillus simplex* P10 Enhances Salt Tolerance in Asparagus by Modulating Amino Acid and Phenylpropanoid Metabolism

**DOI:** 10.3390/plants15121848

**Published:** 2026-06-15

**Authors:** Lingyu Hao, Yingtao Sun, Tao Liu, Lin Meng, Xue Song, Huiling Yan, Yanpo Cao

**Affiliations:** 1 Institute of Cash Crops, Hebei Academy of Agriculture and Forestry Sciences, Shijiazhuang 050051, China; 2 College of Horticulture, Northwest A&F University, Yangling 712100, China; 3 College of Horticulture, Shanxi Agricultural University, Jinzhong 030801, China

**Keywords:** salt stress, asparagus, *Peribacillus simplex*, transcriptome, metabolome

## Abstract

While *Peribacillus simplex* has been reported to alleviate abiotic stress-induced damage in diverse plant species, its precise functional mechanism in mediating salt tolerance in asparagus remains unclear. The present study sought to uncover the molecular regulatory mechanisms through which strain P10 enhances the salt adaptability of asparagus seedlings. We investigated physiological responses, as well as transcriptomic and metabolomic alterations, in P10-inoculated asparagus seedlings grown under saline conditions. The results demonstrated that P10 inoculation alleviated salt-induced physiological damage by enhancing antioxidant enzyme activities and promoting the accumulation of osmotic regulatory substances. Comparative transcriptomic and metabolomic analyses identified 1659 differentially expressed genes (DEGs) and 128 differentially accumulated metabolites (DAMs) between P10-inoculated and non-inoculated seedlings under salt stress. These DEGs were primarily associated with multiple biological pathways, including phenylpropanoid biosynthesis, nitrogen metabolism, and flavonoid biosynthesis pathways (flavone, flavonol, and total flavonoid synthesis). Metabolomic profiling indicated that organic acids constituted the most abundant class of DAMs, followed by amino acids and their derivatives, and flavonoids. Integrated transcriptomic and metabolomic analyses suggested that P10 optimized the amino acid metabolic network under salt stress by upregulating genes involved in nitrogen assimilation, glutathione biosynthesis, and polyamine biosynthesis, thereby promoting amino acid accumulation and enhancing glutathione and polyamine levels. In addition, P10 markedly stimulated flavone and flavonol biosynthesis while maintaining elevated anthocyanin levels. Overall, P10 mitigated salt stress injury in asparagus by regulating amino acid metabolism to improve osmotic balance and growth stability, while simultaneously redirecting phenylpropanoid flux toward flavone and flavonol biosynthetic pathways to fine-tune stress responses.

## 1. Introduction

Soil salinity represents a significant environmental constraint that inhibits plant vegetative growth and reduces agricultural crop yield worldwide. High salinity causes multiple forms of damage to plants, including osmotic imbalance that restricts water uptake, excessive accumulation of sodium and chloride ions that disrupt cellular ion homeostasis, and oxidative damage caused by the overproduction of reactive oxygen species [1,2]. In response to such adverse conditions, plants activate a range of adaptive physiological mechanisms. A well-characterized response is the SOS pathway, which maintains intracellular ionic homeostasis by exporting excess cytoplasmic Na^+^ from plant cells [3]. Concurrently, osmotically active compounds, primarily proline and soluble sugars, accumulate to sustain cellular turgor and ensure proper protein function [4]. The antioxidant defense system, consisting of key enzymes such as superoxide dismutase (SOD), peroxidase (POD), and catalase (CAT), is also markedly activated to scavenge excess reactive oxygen species.

Beyond its fundamental role in growth, nitrogen metabolism plays a critical role in plant stress resilience. Amino acid biosynthesis and catabolism are closely associated with protein synthesis and contribute to osmotic adjustment and redox homeostasis through metabolites such as proline and polyamines [5]. Salt stress typically disrupts amino acid metabolism, resulting in the accumulation of proline and arginine but a reduction in glutamate and branched-chain amino acids, ultimately constraining plant growth [6]. Phenylpropanoid metabolism represents a key secondary metabolic pathway that supplies precursors for flavonoid, flavonol, and anthocyanin biosynthesis [7]. These metabolites are essential for antioxidant defense and stress-related signaling processes [8].

Plant growth-promoting rhizobacteria (PGPR) are widely recognized for their ability to enhance plant tolerance to various environmental stresses [9,10]. Recent reviews have comprehensively summarized the diverse mechanisms through which PGPR enhance plant salt tolerance. These mechanisms include modulation of hormonal signaling, scavenging of reactive oxygen species through enzymatic and non-enzymatic antioxidant systems, and optimization of ion transport to maintain K^+^/Na^+^ homeostasis [11]. Among these beneficial microorganisms, *Peribacillus simplex* has attracted increasing attention due to its positive effects on disease resistance and stress adaptation in crops [12]. Certain strains of *P. simplex* isolated from saline environments exhibit strong salt tolerance and are capable of producing 1-aminocyclopropane-1-carboxylate deaminase, which reduces endogenous ethylene accumulation induced by environmental stress in plant tissues [12]. In addition, these strains can solubilize phosphate and fix atmospheric nitrogen, thereby enhancing nutrient availability [13]. They also produce indole-3-acetic acid and synthesize exopolysaccharides, which contribute to plant resilience against drought and pathogen attack [14,15,16]. Importantly, other *Peribacillus* species beyond *P. simplex* can also effectively mitigate salt stress. *Peribacillus castrilensis* N3, in combination with mauran exopolysaccharide, upregulates sodium transporter genes and increases osmoprotectant accumulation, thereby enhancing osmotic adjustment and maintaining ionic balance in salt-stressed tomato [17]. These findings suggest that the genus *Peribacillus* has the capacity to regulate amino acid metabolism and phenylpropanoid/phenolic metabolism.

*Asparagus officinalis* L. is a nutritionally valuable vegetable crop with moderate salt tolerance; however, exposure to high salinity significantly inhibits seedling growth, disrupts ion and antioxidant homeostasis, and alters global transcriptional profiles [18,19,20]. Recent transcriptomic profiling of asparagus under salinity revealed significant enrichment of differentially expressed genes associated with plant hormone signal transduction and lignin biosynthesis, identifying key regulators such as *Aux/IAA*, *TCH4*, *COMT*, and *POD* [21]. Nevertheless, the specific regulatory pathways through which *P. simplex* alleviates salt-induced damage and enhances salt tolerance in asparagus remain poorly understood, and the integrated transcriptomic-metabolomic network underlying this process remains largely unresolved. Here, integrated multi-omics analyses were performed to uncover how *P. simplex* P10 improves salt tolerance in asparagus. The findings provide a theoretical basis for the practical deployment of functional microbial inoculants in asparagus production under saline soil conditions.

## 2. Results

### 2.1. P10 Enhances Growth Traits of Asparagus Seedlings Under Salt Stress

Under salt stress, inoculation with strain P10 increased asparagus seedling height, shoot fresh weight, shoot dry weight, root fresh weight, and root dry weight by 13.9%, 9.9%, 24.0%, 22.7%, and 14.3%, respectively, compared with the NS treatment. No significant differences were observed between the P and N treatments under non-saline conditions. These results indicate that P10 exerts a strong growth-promoting effect on asparagus seedlings under salt stress.

### 2.2. Influences of P10 on Physiological Traits in Asparagus Seedlings Under Saline Conditions

**Figure 1:**
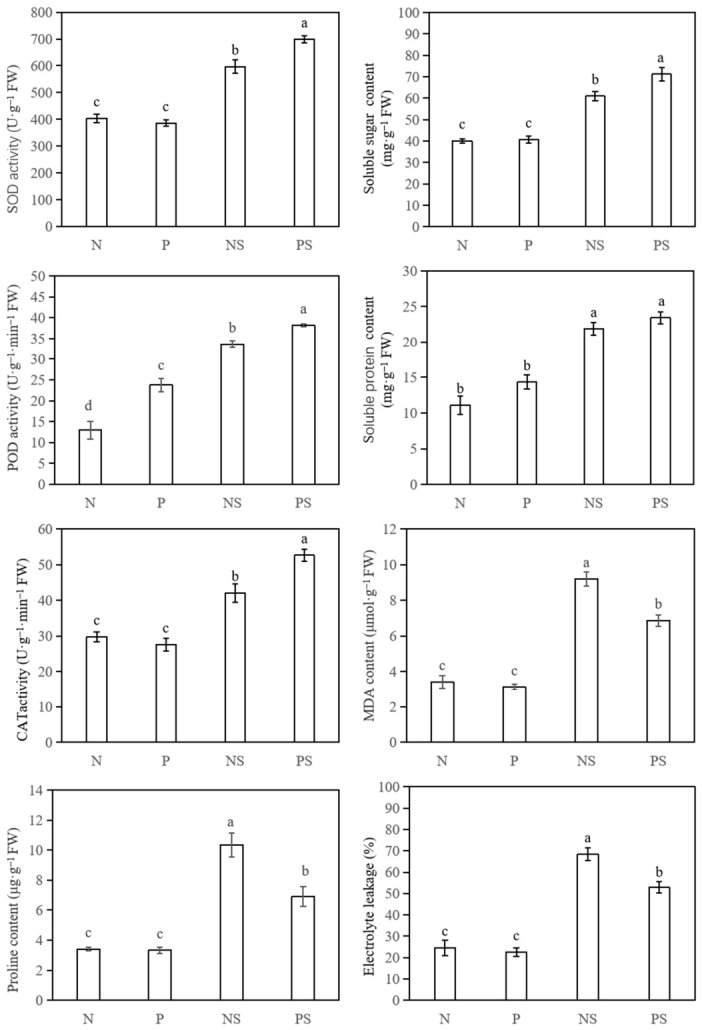
Physiological changes in asparagus seedlings in response to salt stress and inoculation with *Peribacillus simplex* P10. N, distilled water; P, distilled water + P10; NS, NaCl; PS, NaCl + P10. Different lowercase letters within the same subplot indicate significant differences among treatments (*p* < 0.05).

Under non-salt conditions, no significant differences in most physiological traits were found between N and P, except for a prominent rise in POD activity in the P group. In contrast, salt stress induced significant changes in antioxidant enzyme activities in asparagus seedlings (Figure 1). Relative to N, the activities of SOD, POD, and CAT in the NS treatment increased by 47.2%, 150.2%, and 41.6%, respectively. The contents of soluble protein and soluble sugar also increased by 89.5% and 51.2%, respectively. Proline accumulation in the NS group was 94.3% higher than that in the N group. Meanwhile, malondialdehyde (MDA) content and electrolyte leakage increased by 173.2% and 173.6%, respectively.

Inoculation with P10 further enhanced the antioxidant defense capacity of asparagus seedlings under salt stress (Figure 1). Compared with the NS treatment, the PS group showed increases of 18.4%, 16.7%, and 25.6% in SOD, POD, and CAT activities, respectively. The levels of soluble protein and soluble sugar increased by 11.4% and 18.5%, respectively, while proline content exhibited a further increase of 51.5% relative to the NS group. In contrast, MDA content and electrolyte leakage decreased by 24.1% and 24.9%, respectively.

### 2.3. Transcriptional Profiling Analysis

#### 2.3.1. Sequencing Data Summary of Transcriptional Profiling

To elucidate the molecular regulatory mechanisms of strain P10 in salt-stressed asparagus roots, 12 root samples underwent transcriptome sequencing, yielding a total of 526 million raw paired-end reads. After stringent quality filtering and removal of low-quality reads, 57.61 Gb of high-quality clean data were obtained, with Q30 values exceeding 92.13%. The alignment rate to the asparagus reference genome ranged from 93.48% to 94.50%, indicating high data reliability. Twelve DEGs were randomly selected for qRT-PCR validation, and their expression patterns were highly consistent with the transcriptomic results, further confirming the accuracy and reliability of the sequencing data (Figure S1).

#### 2.3.2. Screening of DEGs

**Figure 2:**
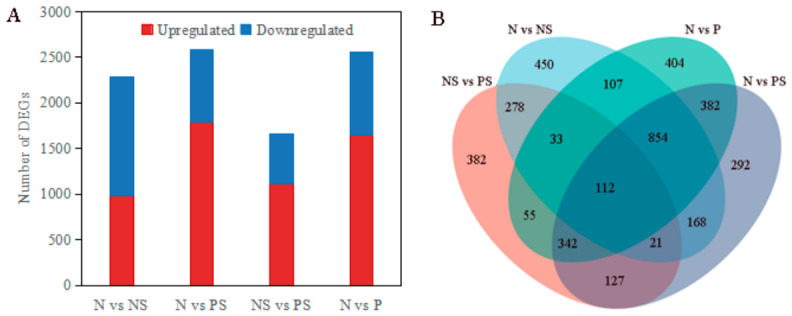
Overview of transcriptomic alterations induced by salt stress and P10 treatment in asparagus. (**A**) Numbers of upregulated and downregulated DEGs in each pairwise comparison. (**B**) Venn diagram illustrating shared and unique DEGs among all experimental conditions.

We identified 2287 DEGs (986 upregulated and 1301 downregulated) in N vs. NS; 2584 DEGs (1795 upregulated and 789 downregulated) in N vs. PS; 1659 DEGs (1111 upregulated and 548 downregulated) in NS vs. PS; and 2556 DEGs (1654 upregulated and 902 downregulated) in N vs. P. Under salt stress alone, a greater number of genes were downregulated, whereas P10 inoculation resulted in a predominance of upregulated genes (Figure 2A,B). This observation indicates that P10 strongly reprograms the transcriptomic profile of asparagus under saline conditions. Although 2556 DEGs were detected in the N vs. P comparison, this comparison was not further analyzed because P10 inoculation produced no observable phenotypic changes under normal conditions (Figure 1, Table 1).

#### 2.3.3. Enrichment Analysis Based on GO and KEGG Databases

**Figure 3:**
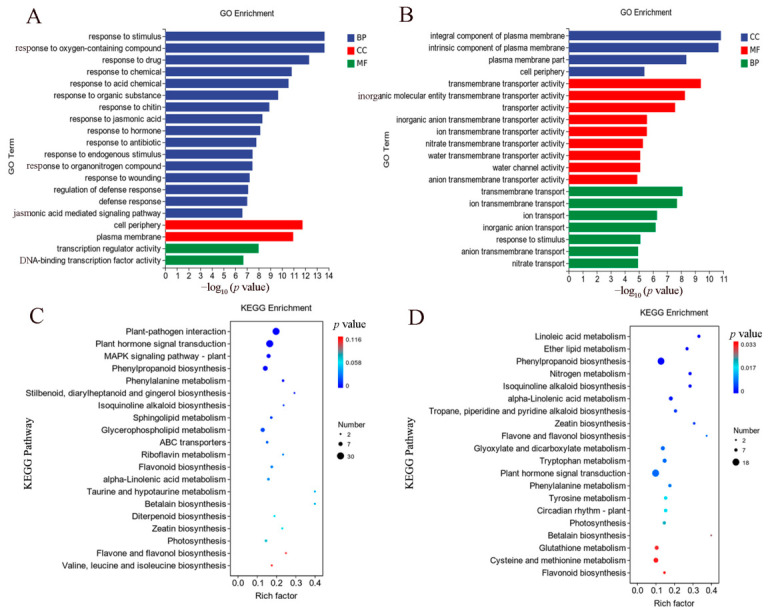
Functional classification of DEGs under salinity with or without P10 treatment. (**A**,**C**) GO terms and KEGG pathways enriched in the N vs. NS comparison. (**B**,**D**) Corresponding enrichments in the NS vs. PS comparison.

In the N vs. NS comparison, DEGs were primarily annotated to biological process (BP) categories, including response to stimulus and response to oxygen-containing compounds; cellular component (CC) categories, such as cell periphery and plasma membrane; and molecular function (MF) categories, including transcription regulator activity and DNA-binding transcription factor activity (Figure 3A). In the NS vs. PS comparison, DEGs were significantly enriched in CC terms associated with intrinsic components of the plasma membrane; MF terms including transmembrane transporter activity, ion transmembrane transporter activity, water channel activity, and nitrate transmembrane transporter activity; and BP terms related to ion transmembrane transport and nitrate transport (Figure 3B). These findings suggest that P10 primarily modulates ion and water transport processes under salt stress.

In the N vs. NS group, enriched KEGG pathways mainly included plant–pathogen interaction, plant hormone signal transduction, MAPK signaling pathway, and phenylpropanoid biosynthesis (Figure 3C). In contrast, DEGs in the NS vs. PS comparison were predominantly enriched in phenylpropanoid biosynthesis, nitrogen metabolism, flavone and flavonol biosynthesis, flavonoid biosynthesis, and glutathione metabolism (Figure 3D).

### 2.4. Metabolite Identification and DAM Screening

**Figure 4:**
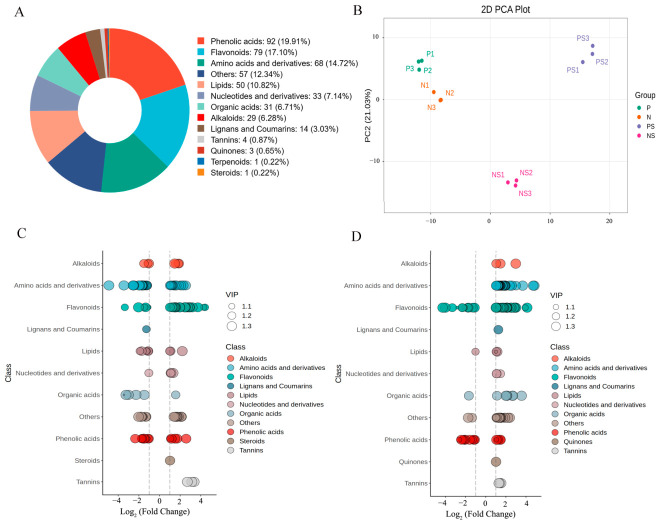
Metabolic profiles and differential metabolite accumulation across the four treatments. (**A**) Categories of detected metabolites. (**B**) PCA plot showing sample clustering. (**C**,**D**) Volcano plots of DAMs in N vs. NS and NS vs. PS comparisons, respectively.

Changes in metabolic profiles regulated by strain P10 were investigated using a widely targeted metabolomics approach. In total, 621 metabolites were detected across all root samples (Figure 4A), including phenolic acids (19.91%), flavonoids (17.1%), amino acids and derivatives (14.72%), others (12.34%), lipids (10.82%), nucleotides and derivatives (7.14%), organic acids (6.71%), alkaloids (6.28%), lignans and coumarins (3.03%), tannins (0.87%), quinones (0.65%), terpenoids (0.22%), and steroids (0.22%). Principal component analysis (PCA) revealed clear separation among the four treatments, with tight clustering of biological replicates. The first two principal components, PC1 and PC2, explained 39.3% and 21.03% of the total variance, respectively (Figure 4B).

A total of 119 DAMs were identified in N vs. NS, and 115 DAMs in N vs. PS. In the NS vs. PS comparison, 128 DAMs were detected, while 107 DAMs were identified in N vs. P (Table S2 and Figure 4C,D). Compared with the NS treatment, the PS group exhibited increased accumulation of 23 amino acids and derivatives, six saccharides and alcohols, seven organic acids, and 18 flavonoids. In contrast, these metabolites either decreased or remained unchanged in the NS group relative to the N group. Additionally, eight amino acids, seven sugars, and 10 flavonoids were upregulated in both N vs. NS and NS vs. PS comparisons (Table S2).

**Figure 5:**
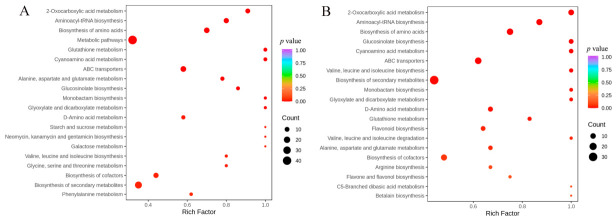
KEGG pathway enrichment analysis of DAMs under salt stress and P10 treatment. (**A**) N vs. NS comparison. (**B**) NS vs. PS comparison.

Metabolites identified in the N vs. NS comparison were mainly associated with amino acid biosynthesis, valine, leucine, and isoleucine biosynthesis, glutathione metabolism, and flavonoid biosynthesis pathways (Figure 5A). In the NS vs. PS group, the altered metabolites were significantly enriched in arginine biosynthesis, as well as alanine, aspartate, and glutamate metabolism pathways (Figure 5B).

### 2.5. Integrated Transcriptomic and Metabolomic Analysis

Based on combined transcriptomic and metabolomic datasets, we focused on pathways associated with amino acid metabolism, phenylpropanoid metabolism, and flavonoid biosynthesis.

#### 2.5.1. Amino Acid Metabolism in P10-Inoculated Asparagus Under Salt Stress

**Figure 6:**
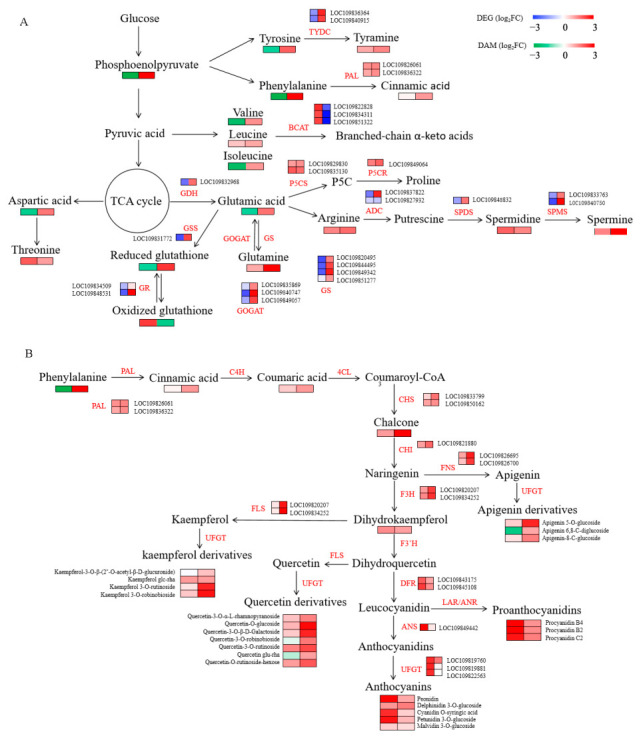
Integrated transcriptomic and metabolomic analysis of key pathways regulated by P10 in salt-stressed asparagus. (**A**) Coordinated changes in genes and metabolites in amino acid metabolism. (**B**) Coordinated changes in phenylpropanoid and flavonoid metabolism. Heatmaps represent log_2_ fold-change (log_2_FC) values for each comparison (N vs. NS, left panel; NS vs. PS, right panel). For genes, blue indicates downregulation and red indicates upregulation. For metabolites, green indicates decreased levels and red indicates increased levels. Arrows indicate the inferred directional progression of metabolic processes in the pathways.

Salt stress markedly altered amino acid metabolism in asparagus (Figure 6A). Under salt stress alone, three glutamate synthase (*GOGAT*) genes, four glutamine synthetase (*GS*) genes, one glutamate dehydrogenase (*GDH*) gene, and two tyrosine decarboxylase (*TYDC*) genes were downregulated, accompanied by decreased levels of glutamate, phenylalanine, and tyrosine. In contrast, two Δ^1^-pyrroline-5-carboxylate synthase (*P5CS*) genes and one Δ^1^-pyrroline-5-carboxylate reductase (*P5CR*) gene were upregulated, resulting in increased proline accumulation. Three branched-chain amino acid transaminase (*BCAT*) genes were upregulated, whereas the endogenous levels of leucine, isoleucine, and valine declined. In the polyamine pathway, two arginine decarboxylase (*ADC*) genes, one spermidine synthase (*SPDS*) gene, and two spermine synthase (*SPMS*) genes were downregulated, although spermidine and spermine levels increased. In the glutathione pathway, two glutathione reductase (*GR*) genes and one glutathione synthetase (*GSS*) gene were upregulated. However, oxidized glutathione (GSSG) levels increased significantly, whereas reduced glutathione (GSH) levels decreased significantly (Table S3).

Following P10 inoculation, the expression of *GOGAT*, *GS*, *GDH*, *TYDC*, and phenylalanine ammonia-lyase (*PAL*) genes was significantly upregulated, resulting in increased levels of glutamate, phenylalanine, and tyrosine. *P5CS* remained upregulated, whereas *BCAT* expression was downregulated, consistent with elevated proline and branched-chain amino acid levels. Furthermore, genes involved in polyamine and glutathione biosynthesis (*ADC*, *SPDS*, *SPMS*, *GR*, and *GSS*) were upregulated, promoting the accumulation of spermidine, spermine, and GSH. These results indicate that P10 enhances salt tolerance by restoring nitrogen assimilation, promoting the synthesis of proline, polyamines, and glutathione, balancing branched-chain amino acid metabolism, and optimizing the overall amino acid network (Table S3).

#### 2.5.2. Phenylpropanoid Metabolism in P10-Inoculated Asparagus Under Salt Stress

Salt stress activated phenylpropanoid and flavonoid metabolism in asparagus (Figure 6B), resulting in increased levels of cinnamic acid, chalcones, dihydroquercetin, proanthocyanidins, and anthocyanins. Transcriptomic analysis showed the upregulation of two PAL genes, two chalcone synthase (*CHS*) genes, one chalcone isomerase (*CHI*) gene, and two flavanone 3-hydroxylase (*F3H*) genes under salt stress. Two dihydroflavonol 4-reductase (*DFR*) genes and three UDP-glucose:flavonoid glycosyltransferase (*UFGT*) genes involved in anthocyanin biosynthesis were strongly induced in the root tissues.

Following P10 inoculation, phenylpropanoid metabolic flux was reprogrammed. Compared with salt stress alone, anthocyanin and dihydroquercetin levels increased only modestly, whereas apigenin, kaempferol, and quercetin derivatives accumulated at substantially higher levels. At the transcriptional level, P10 strongly upregulated flavonol synthase (*FLS*) and flavone synthase (*FNS*) genes in the flavonol branch, while the expression levels of *PAL*, *CHS*, *CHI*, and *F3H* remained relatively stable (Table S4). These findings suggest that asparagus primarily activates the anthocyanin branch for stress defense under salt stress, whereas P10 redirects metabolic flux toward flavone and flavonol biosynthesis, supporting a more sustained defense strategy *coupled* with growth maintenance.

## 3. Discussion

Salt stress represents a major constraint on agricultural productivity and crop growth. Earlier studies have shown that *P. simplex* can alleviate salt-induced damage in crops [22]. Physiological analyses in the present study confirmed that strain P10 activates antioxidant defense systems and enhances osmotic regulation capacity in asparagus seedlings, thereby mitigating growth inhibition caused by salinity. Integrated transcriptomic and metabolomic analyses further revealed that P10 reshapes metabolic reprogramming in amino acid metabolism, phenylpropanoid pathways, and flavonoid biosynthesis in asparagus under salt stress.

### 3.1. Modulation of Amino Acid Metabolic Pathway by P10 Under Saline Conditions

The amino acid metabolic network plays a central role in plant adaptation to abiotic stress [5]. Salinity stress often disrupts intracellular amino acid homeostasis and inhibits nitrogen assimilation [23]. Physiological and molecular evidence from this study demonstrated that salt treatment suppressed the expression of key nitrogen assimilation genes, including *GOGAT*, *GS*, and *GDH*, in asparagus seedlings, accompanied by decreased levels of glutamate, phenylalanine, and tyrosine, indicating reduced nitrogen assimilation capacity. However, elevated expression of *P5CS*, together with increased accumulation of proline, arginine, and glutamine, enabled asparagus to adapt to osmotic stress [24]. Salt stress also increased endogenous spermidine and spermine levels in asparagus seedlings as a potential defense strategy, despite transcriptional downregulation of their biosynthetic genes. This discrepancy may be attributed to sufficient precursor availability or reduced catabolic activity rather than transcriptional regulation [25]. Increased *BCAT* expression, coupled with decreased branched-chain amino acid levels, suggests enhanced catabolism to provide carbon skeletons for energy metabolism [5].

PGPR typically promote plant growth by improving nitrogen utilization and activating secondary metabolic pathways [26,27]. In the present study, P10 inoculation significantly upregulated the expression of *GOGAT*, *GS*, and *GDH* in asparagus seedlings, restoring nitrogen assimilation capacity and increasing the levels of multiple amino acids. This effect further enhanced *P5CS* expression and promoted proline accumulation, thereby strengthening osmotic protection. Upregulation of *GSH* biosynthesis genes facilitated the accumulation of stress-resistant metabolites such as *GSH* in asparagus seedlings [28]. In addition, P10 treatment downregulated *BCAT* expression, reducing the degradation of branched-chain amino acids and preserving substrates for protein synthesis. Collectively, P10 reconfigured the nitrogen metabolic network of asparagus seedlings to coordinate stress defense and growth homeostasis. These findings are consistent with previous reports of PGPR-regulated nitrogen metabolism in other crops and demonstrate, for the first time, that *P. simplex* P10 reconfigures the nitrogen metabolic network in salt-stressed asparagus [27,28].

### 3.2. P10 Shifts Phenylpropanoid Flux to Optimize Defense Strategy

Phenylpropanoid metabolites perform diverse physiological functions in plant stress adaptation [29]. Anthocyanins effectively scavenge excess reactive oxygen species under salinity, whereas flavones and flavonols contribute to long-term antioxidant capacity and participate in signaling regulation [30]. Transcriptomic analysis revealed that salt stress induced the expression of core phenylpropanoid pathway genes, particularly the key anthocyanin biosynthetic gene *DFR*, resulting in substantial anthocyanin accumulation. This anthocyanin-dominated defense mechanism represents a common adaptive response in plants under salt stress [31].

Following P10 inoculation, *FLS* and *FNS* genes in the flavonol branch were significantly upregulated, leading to increases in kaempferol, quercetin, and apigenin derivative levels, whereas anthocyanin accumulation increased only modestly but remained elevated. This shift suggests that P10 may modulate upstream MYB transcription factors and phytohormonal signals, which are known to differentially regulate flavonoid branches under salt stress [32]. Flavonoids function as antioxidants, signaling molecules, and regulators of auxin transport, and they are relatively less energetically costly to synthesize [33,34,35]. On a per-carbon basis, flavonols require lower biosynthetic investment than anthocyanins, reflecting a carbon-defense trade-off that conserves energy for growth under stress [36]. They also facilitate beneficial microbial colonization [37]. PGPR have been reported to coordinate photosynthesis and metabolism to enhance stress tolerance [38], and plant growth-promoting *Bacillus* species have been shown to improve salt tolerance in multiple crops [39,40]. Therefore, P10 redirects phenylpropanoid flux from anthocyanin-mediated defense toward a more balanced flavonoid-based strategy that supports both stress tolerance and plant growth, thereby functioning as a molecular switch that optimizes defense allocation. Notably, P10 inoculation simultaneously upregulated genes involved in nitrogen assimilation and phenylpropanoid metabolism, suggesting potential crosstalk between these pathways, which has not been extensively addressed in previous PGPR studies [41].

This study reveals a previously unrecognized metabolic mechanism underlying P10-conferred salt tolerance, thereby expanding current understanding of plant-PGPR interactions. In addition, it supports the development of P10-based bioinoculants for asparagus cultivation in saline soils, offering a sustainable strategy for agriculture on marginal lands.

## 4. Materials and Methods

### 4.1. Experimental Materials and Design

The asparagus cultivar used in this study was ‘Jiyu Green 2’, provided by the Hebei Academy of Economic Crops. The bacterial strain used was *P. simplex* P10 (GenBank No. MT878549), isolated and identified by the Hebei Institute of Soil and Fertilizer, with a spore density of 1.0 × 10^9^ CFU·g^−1^. The phenotypic and biochemical characteristics of this strain were described by the original isolators [42]. The growth substrate consisted of perlite, vermiculite, and peat mixed at a volume ratio of 1:2:1 and was sterilized by dry heat at 160 °C for 4 h prior to use. The sterilized substrate was divided into two portions: one was thoroughly mixed with the active P10 inoculant, while the other was combined with an equal amount of inactivated inoculant that had been autoclaved at 121 °C for 30 min to eliminate bacterial activity. The final inoculation rate was 1 g of inoculant per pot containing 600 g of substrate, and the mixture was transferred into plastic pots (12 cm in diameter).

Surface-sterilized asparagus seeds were sown directly into the pots, with one plant per container. Seedlings were cultivated in a controlled environment chamber under a 12/12 h photoperiod (light/dark), temperature settings of 28 °C (light) and 18 °C (dark), and relative humidity of 70–80%. After 40 days, uniform and healthy seedlings were selected for salt treatment. For salt stress application, 200 mL of 200 mM NaCl solution was applied to each pot, whereas control plants received distilled water. The selection of 200 mM NaCl and a seven-day treatment period was based on our previous systematic evaluation of salt tolerance in asparagus germplasm at different growth stages [16], which validated these conditions as suitable for mechanistic studies. Four treatments were established: (1) Control (N): distilled water + inactivated bacteria; (2) Salt stress (NS): NaCl + inactivated bacteria; (3) Only active bacteria (P): distilled water + active P10; and (4) Salt stress + active bacteria (PS): NaCl + active P10. Each treatment included three independent biological replicates. A total of 12 root samples were collected (4 treatments × 3 biological replicates), with each biological replicate comprising a pool of three seedlings. Root tissues were collected seven days after salt treatment, immediately frozen in liquid nitrogen, and stored at −80 °C for subsequent analyses. All 12 samples were processed individually for physiological, transcriptomic, and metabolomic analyses.

### 4.2. Determination of Growth and Physiological Characteristics

Plant height was measured with a ruler. Immediately after collection, the fresh weights of shoots and roots were recorded, and then the samples were dried to constant weight for dry weight determination. Physiological parameters of asparagus roots were measured using commercial assay kits, except for electrolyte leakage. The evaluated parameters included the activities of SOD, POD, and CAT, as well as the contents of MDA, soluble protein, soluble sugar, and proline. All assay kits were obtained from Boxbio Science & Technology Co., Ltd. (Beijing, China), and all procedures were conducted strictly according to the manufacturer’s instructions. Electrolyte leakage was determined by measuring the relative conductivity of root extracts using a conductivity meter.

### 4.3. Transcriptome Sequencing and Analysis

Root samples (12 in total) were homogenized in TRIzol reagent (Invitrogen, Carlsbad, CA, USA) for total RNA extraction according to the manufacturer’s protocol. High-quality RNA samples were randomly fragmented and reverse-transcribed to synthesize first-strand cDNA for library construction. The resulting libraries were diluted to a final concentration of 1.8 nM, after which 10 µL of the diluted library was used for sequencing on the Illumina NovaSeq 6000 platform (Illumina, San Diego, CA, USA).

Raw sequencing reads were processed using Fastp (v0.22.0) for quality control, including adapter trimming and removal of low-quality reads using a Q20 quality threshold. Reads shorter than 50 bp were discarded to generate clean data. The clean reads were then aligned to the asparagus reference genome (GCF_001876935.1) with HISAT2 (v2.2.1). HTSeq (v0.9.1) was employed to calculate gene read counts, and gene expression levels were normalized as fragments per kilobase of transcript per million mapped reads (FPKM). Differential expression analysis was performed using DESeq (v1.38.3), with thresholds of |log_2_FC| > 1 and adjusted *p*-value (padj) < 0.05. Gene Ontology (GO) and Kyoto Encyclopedia of Genes and Genomes (KEGG) enrichment analyses were subsequently conducted for the identified DEGs. Twelve DEGs were randomly selected for qRT-PCR validation to confirm the reliability of the transcriptomic data. The qRT-PCR procedures followed the protocol described by Zhang et al. [13], and all primer sequences are provided in Table S1.

### 4.4. Widely Targeted Metabolomic Analysis

Twelve root samples were thoroughly ground in liquid nitrogen. For each sample, 100 mg of homogenized powder was extracted with 1.2 mL of 70% methanol and incubated overnight at 4 °C, followed by centrifugation at 10,000 rpm for 10 min at 4 °C. The resulting supernatant was filtered through a 0.22 μm membrane and subsequently analyzed by UPLC-MS/MS. The UPLC system consisted of a Shim-pack UFLC SHIMADZU CBM30A system (Shimadzu, Kyoto, Japan), coupled to a 6500 Q TRAP mass spectrometer (Applied Biosystems, Foster City, CA, USA). The detailed analytical method is provided in File S1. Metabolites were annotated using the MetWare database based on secondary mass spectrometry data, and relative quantification was performed in multiple reaction monitoring mode. PCA was used to evaluate overall metabolic variation among samples, whereas orthogonal partial least squares discriminant analysis (OPLS-DA) was applied to distinguish differences between treatment groups. DAMs were identified using thresholds of VIP ≥ 1 and FC ≥ 2 or ≤0.5.

### 4.5. Statistical Analysis

All statistical analyses were performed using SPSS 22.0. Physiological and molecular parameters were compared among treatments using one-way ANOVA, followed by Duncan’s multiple range test at *p* < 0.05.

## 5. Conclusions

This study combined physiological analyses with transcriptomic and metabolomic profiling to elucidate the molecular mechanisms by which *Peribacillus simplex* P10 alleviates salt stress in asparagus. P10 inoculation significantly enhanced antioxidant capacity and osmotic adjustment, reduced membrane lipid peroxidation, and optimized amino acid metabolism by promoting nitrogen assimilation, glutathione biosynthesis, and polyamine production. Furthermore, P10 reprogrammed phenylpropanoid metabolism by shifting the defense strategy from anthocyanin-dependent responses to sustained flavonoid- and flavonol-mediated homeostasis. Overall, P10 alleviates salt stress injury by modulating physiological and metabolic pathways in asparagus. These findings provide a valuable theoretical basis for the development and application of microbial inoculants in salt-tolerant asparagus cultivation.

## Figures and Tables

**Table 1:** Growth parameters of asparagus seedlings under different treatments.

Treatment	Plant Height /cm	Aboveground Fresh Weight /g	Root Fresh Weight /g	Aboveground Dry Weight /g	Root Dry Weight /g
N	52.42 ± 0.52 a	35.53 ± 0.51 a	23.85 ± 0.59 a	4.56 ± 0.33 a	4.21 ± 0.33 a
P	53.36 ± 0.63 a	34.71 ± 0.77 a	24.05 ± 0.82 a	5.01 ± 0.33 a	4.35 ± 0.33 a
NS	35.36 ± 0.41 c	23.85 ± 1.02 c	19.01 ± 0.47 c	3.21 ± 0.33 c	3.56 ± 0.33 c
PS	40.28 ± 0.55 b	26.21 ± 0.41 b	23.33 ± 0.38 b	3.98 ± 0.33 b	4.07 ± 0.33 b

Note: Different lowercase letters within the same column indicate significant differences among treatments (*p* < 0.05). N, distilled water; P, distilled water + P10; NS, NaCl; PS, NaCl + P10.

## Data Availability

Raw RNA sequencing data are available from the NGDC repository (https://ngdc.cncb.ac.cn/) under accession PRJCA054206.

## Supplementary Materials

The following supporting information can be downloaded at: https://www.mdpi.com/article/10.3390/plants15121848/s1, Figure S1: qRT-PCR validation of eight selected DEGs; File S1: Detailed UPLC-ESI-MS/MS analytical conditions; Table S1: Primer sequences used for qRT-PCR analysis; Table S2: DAMs in each comparison; Table S3: DEGs and DAMs associated with amino acid metabolism; Table S4: DEGs and DAMs involved in phenylpropanoid and flavonoid metabolism.
