# Supplementary material for: Peribacillus simplex P10 Enhances Salt Tolerance in Asparagus by Modulating Amino Acid and Phenylpropanoid Metabolism
Source: Plants (Basel). 2026 Jun 15;15(12):1848. doi: 10.3390/plants15121848 (PMC13306513; doi:10.3390/plants15121848)
Supplement: Supplementary file 1 [file plants-15-01848-s001.zip › Figure S1.pdf]

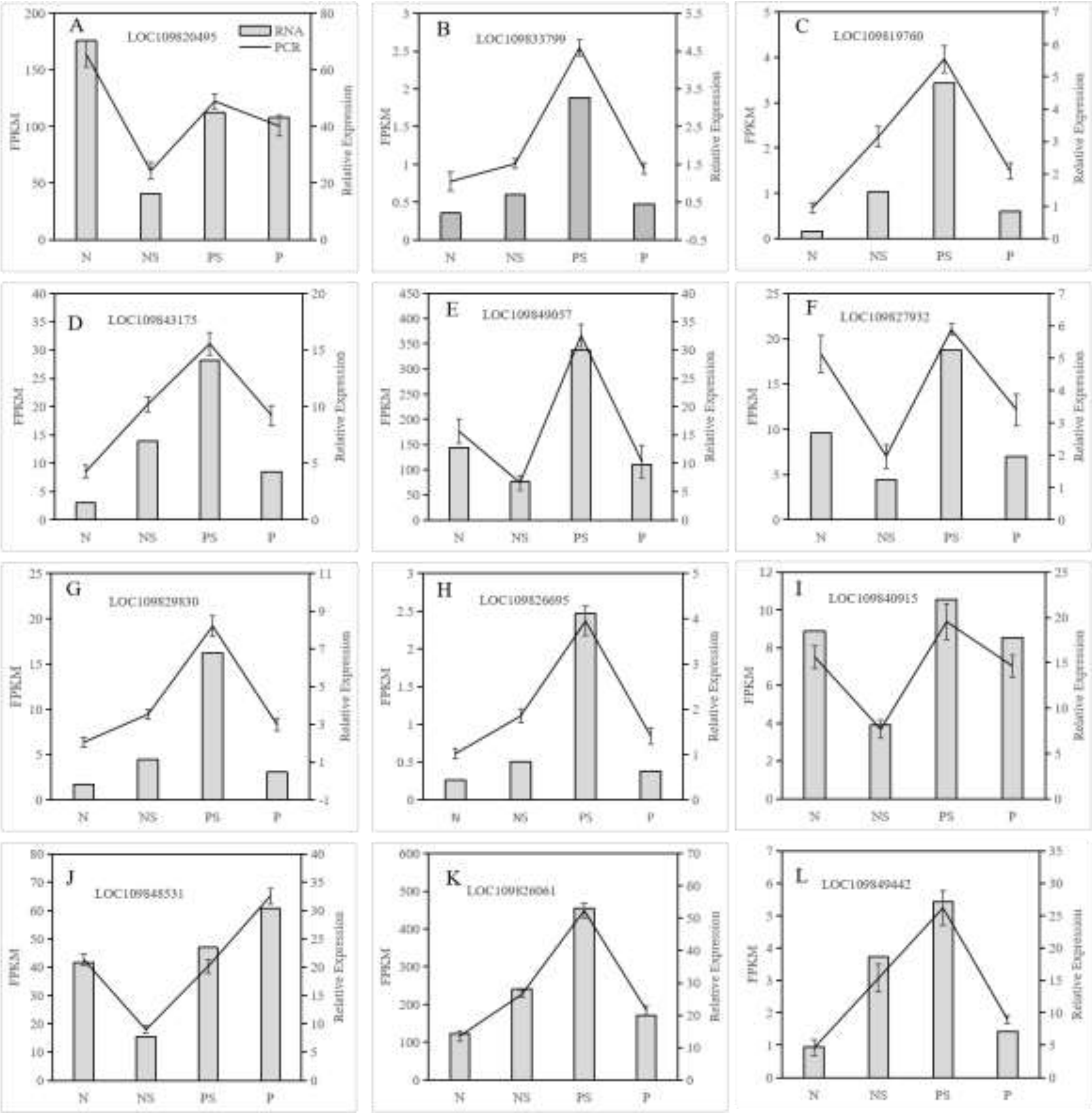

Figure S1. qRT-PCR validation of eight selected DEGs. (A) glutamine synthetase. (B) chalcone synthase. (C) UDP-glucose:flavonoid glycosyltransferase. (D) dihydroflavonol 4-reductase. (E) glutamate synthase. (F) arginine decarboxylase. (G) Δ^1-pyrroline-5-carboxylate synthase. (H) flavone synthase. (I) tyrosine decarboxylase. (J) glutathione reductase. (K) phenylalanine ammonia-lyase. (L) anthocyanidin synthase.
