# Supplementary material for: Peribacillus simplex P10 Enhances Salt Tolerance in Asparagus by Modulating Amino Acid and Phenylpropanoid Metabolism
Source: Plants (Basel). 2026 Jun 15;15(12):1848. doi: 10.3390/plants15121848 (PMC13306513; doi:10.3390/plants15121848)
Supplement: Supplementary file 1 [file plants-15-01848-s001.zip › File S1.pdf]

File S1. Detailed UPLC-ESI-MS/MS analytical conditions

The UPLC-ESI-MS/MS analysis was performed using a Shim-pack UFLC SHIMADZU CBM30A system (Shimadzu, Kyoto, Japan) equipped with a Waters ACQUITY UPLC HSS T3 C18 column (1.8 μm, 2.1 mm × 100 mm; Waters, Milford, MA, USA). The column temperature was maintained at 40 °C, and the injection volume was 2 μL. The mobile phase consisted of solvent A (pure water with 0.04% acetic acid) and solvent B (acetonitrile with 0.04% acetic acid) at a flow rate of 0.4 mL/min. The gradient program was as follows: 0–10 min, linear gradient from 95% A / 5% B to 5% A / 95% B; 10–11 min, hold at 5% A / 95% B; 11–11.1 min, return to 95% A / 5% B; 11.1–14 min, hold at 95% A / 5% B. Mass spectrometry was performed on an API 6500 Q TRAP mass spectrometer (Applied Biosystems, Foster City, CA, USA) equipped with an electrospray ionization (ESI) Turbo IonSpray interface. Data were acquired in positive and negative ion modes using multiple reaction monitoring (MRM). The ESI source parameters were set as follows: ion spray voltage, +5500 V (positive mode) and –4500 V (negative mode); source temperature, 550 °C; ion source gas 1 (GS1), 50 psi; ion source gas 2 (GS2), 60 psi; curtain gas (CUR), 30 psi; collision gas (CAD), high. For MRM experiments, nitrogen was used as the collision gas at a pressure of 5 psi, and declustering potential (DP) and collision energy (CE) were optimized for each MRM transition. Data acquisition and processing were performed using Analyst 1.6.3 software (AB Sciex).
